# Health care system costs related to potentially inappropriate medication use involving opioids in older adults in Canada

**DOI:** 10.1186/s12913-023-10303-2

**Published:** 2023-11-24

**Authors:** Carina D’Aiuto, Carlotta Lunghi, Line Guénette, Djamal Berbiche, Karine Bertrand, Helen-Maria Vasiliadis

**Affiliations:** 1 Department of Community Health Sciences, Faculty of Medicine and Health Sciences, University of Sherbrooke (Longueuil campus), 150 Place Charles-Le Moyne, Longueuil, QC J4K 0A8 Canada; 2 Charles-Le Moyne Research Center (CR-CLM), 150 Place Charles-Le Moyne, Longueuil, QC J4K 0A8 Canada; 3 Department of Health Sciences, Université du Québec à Rimouski (Lévis campus), 1595 Boulevard Alphonse-Desjardins, Lévis, QC G6V 0A6 Canada; 4 Population Health and Optimal Health Practices Research Unit, CHU de Québec Research Center, 1050 Chemin Sainte-Foy, Québec, QC G1S 4L8 Canada; 5 Department of Medical and Surgical Sciences, University of Bologna, Via Zamboni, 33, 40126 Bologna, BO Italy; 6 Faculty of Pharmacy, Laval University, 1050 Av. de La Médecine, Québec City, QC G1V 0A6 Canada

**Keywords:** Cost analysis, Drug utilization, Elderly, Health care costs, Pain

## Abstract

**Background:**

Older adults are at risk of potentially inappropriate medication use given polypharmacy, multimorbidity, and age-related changes, which contribute to the growing burden associated with opioid use. The objective of this study was to estimate the costs of health service utilization attributable to opioid use and potentially inappropriate medication use involving opioids in older adults in a public health care system.

**Methods:**

The sample included 1201 older adults consulting in primary care, covered by the public drug plan, without a cancer diagnosis and opioid use in the year before interview. Secondary analyses were conducted using two data sources: health survey and provincial administrative data. Health system costs included inpatient and outpatient visits, physician billing, and medication costs. Unit costs were calculated using annual financial and activity reports from 2013–2014, adjusted to 2022 Canadian dollars. Opioid use and potentially inappropriate medication use involving opioids were identified over 3 years. Generalized linear models with gamma distribution were employed to model 3-year costs associated with opioid use and potentially inappropriate medication use involving opioids. A phase-based approach was implemented to provide descriptive results on the costs associated with each phase: i) no use, ii) opioid use, and iii) potentially inappropriate medication use involving opioids.

**Results:**

Opioid use and potentially inappropriate medication use involving opioids were associated with adjusted 3-year costs of $2,222 (95% CI: $1,179-$3,264) and $8,987 (95% CI: $7,370-$10,605), respectively, compared to no use. In phase-based analyses, costs were the highest during inappropriate use.

**Conclusions:**

Potentially inappropriate medication use involving opioids is associated with higher costs compared to those observed with opioid use and no use. There is a need for more effective use of health care resources to reduce costs for the health care system.

**Supplementary Information:**

The online version contains supplementary material available at 10.1186/s12913-023-10303-2.

## Background

The societal economic burden associated with opioid use in Canada was $5.9 billion in 2017, mostly driven by losses in productivity, health care costs including physician time, prescription drugs, and hospitalizations [1]. In fact, a report showed the highest rates of hospitalizations (20.1 hospitalizations per 100,000 population) associated with opioid poisonings in those aged 65 and older compared to other age groups [2]. Accidental opioid poisonings were the most common reason for hospitalization in older adults [2]. This is likely due to the increased risk of adverse drug reactions in older adults given high rates of polypharmacy, multimorbidity, and age-related changes [3], which all contribute to the growing opioid use problem in this population.

With the aging population and the increase in pain and concomitant health conditions that require treatment, older adults are at an increased risk of potentially inappropriate medication use involving opioids (PIOU), which has been associated with adverse health effects, increased health service utilization [4–6] and increased health system costs [4, 5, 7–9].

The rare studies evaluating the costs associated with PIOU in older adults have shown excess costs ranging between $1,526 and $4,976 US annually in the American health care context [10–12]. However, the validity of these studies is limited given that only some controlled for mental health comorbidities [10, 11] and none controlled for substance use disorders. Studies controlling for mental health conditions, however, were conducted in populations with specific clinical profiles relating to pain conditions [10, 11]. It should also be noted that these studies assessed the presence of drug interactions involving opioids, therefore constituting a specific clinical context [10, 11]. Moreover, none of the studies assessed whether costs differed by sex, despite that older females consistently show higher risk of PIOU compared to older males [13–22]. There is therefore a need to improve upon the estimates of costs associated with PIOU in older adults by defining costs within a Canadian public health care system context. Furthermore, studies in the current literature are not consistent in defining PIOU. This highlights a need to use a more reliable and complete definition of PIOU in older adults, such as the complete Beers definition which identifies specific opioids that should be avoided due to lack of effectiveness and high risk of neurotoxicity, as well as in combination with other medications due to a risk of drug-drug interactions (such as benzodiazepines, gabapentin, pregabalin, and other central nervous system medications) or in individuals with a history of falls/fractures [23]. Finally, some of the previous studies may also be at risk for immortal time bias [24] given that the period between the start of follow-up and the first PIOU was not properly accounted for [10, 11].

In light of the current literature, the objective of this study was to evaluate the costs related to health service utilization associated with opioid use and PIOU over a 3-year period and to provide descriptive analyses using a phase-based costing approach [25]. This method provides clinically meaningful cost estimates in the presence of censoring, therefore improving the validity of results by minimizing biases due to loss to follow-up. Furthermore, the present study aimed to minimize the effect of confounding factors in estimating the costs associated with PIOU using inverse probability of treatment weighting (IPTW).

## Methods

### Study design

The *Étude sur la Santé des Aînés* (ESA)-Services longitudinal study recruited community-dwelling older adults consulting in primary care clinics between 2011 and 2013 in Québec, Canada. Older adults aged 65 years and older interested in participating in the study were contacted for an at-home interview. Of the 1,811 older adults interviewed in the ESA-Services study, health administrative data in the 3 years prior and following the baseline interview (T1) were successfully merged to health survey data for 1,657 consenting participants. Further details of the longitudinal study, which aimed to assess health service utilization and inappropriate prescribing in a primary care population of older adults, can be found in a previous article [26]. The analytic sample for this study was limited to 1201 older adults with no malignant tumor diagnosis (ICD-9: 140.X-165.X, 170.X-176.X, 179.X-209.X, ICD-10: C00.X-C26.X, C30.X-C41.X, C43.X-C58.X, C60.X-C97.X) and no opioid use in the year before the at-home interview (T1). Older adults with cancer were excluded given that their health service use is based on specific treatment protocols that are not well documented in administrative databases (ex. chemotherapy, radiotherapy). Individuals with opioid use in the year before the interview were excluded to implement a wash-out period. The sample was also limited to older adults who were covered by the provincial public drug plan over the follow-up, allowing the study of opioid use over time. The province of Québec has a universal health program that covers physician and hospital services for the entire population, as well as a public drug plan that covers virtually all individuals aged 65 years and older for their medications, with more than 90% of older adults covered. As such, data on physician services and drugs dispensed are available from the *Régie de l’assurance maladie du Québec (RAMQ)* and data on hospitalizations are available from the *Maintenance et exploitation des données pour l’étude de la clientèle hospitalière (MED-ÉCHO)*. Ethics approval for this secondary data analysis was obtained from the *CIUSSS Estrie-Centre Hospitalier de Sherbrooke* (CHUS) (# 2021–3807).

### Opioid and potentially inappropriate medication use involving opioids

Opioid use was identified over a 3-year period following interviews using medication claims data from the RAMQ. PIOU was identified over 3 years using the 2019 Beers criteria [23]. The Beers criteria are a list of medications that should be avoided or used with caution in older adults due to a high risk of side effects, adverse reactions, or drug interactions [23]. The most recent Beers criteria, published in 2019, were used to reflect the current impact of PIOU in terms of costs. PIOU included exposure to pentazocine use, meperidine use, concomitant opioid and benzodiazepine use, concomitant opioid and pregabalin or gabapentin use, opioid use in those with a history of falls or fractures, or opioid use combined with two central nervous system (CNS)-active medications. A 1-day overlap in the days’ supply was considered potentially inappropriate for cases of concomitant use. That is, an overlap of an opioid supply by at least one day with the supply of a benzodiazepine, gabapentin, pregabalin, or two other CNS-active medications was deemed potentially inappropriate. The operationalization of this variable has been described previously [15]. Individuals were grouped based on their exposure as no opioid use, opioid use (not potentially inappropriate), or PIOU over 3 years.

Observation time for the descriptive phase-based approach was divided into 3 phases: (i) no opioid use, (ii) opioid use, and (iii) PIOU. Individuals who did not use an opioid in the follow-up period had their entire observation period assigned to phase (i), opioid users contributed a portion of their time to phase (i) until the date that the first opioid was dispensed, after which the remainder of the follow-up period was attributed to phase (ii). Finally, individuals with PIOU contributed to all 3 phases, unless their first opioid use was inappropriate, in which case they would only contribute to phases (i) and (iii). Observation time ended at the time of death or the end of the 3-year follow-up. In this way, exposure was defined using a hierarchical approach such that an individual cannot return to a previous phase once they have entered a new phase under the assumption that returning to baseline health care utilization within the study period after having experienced a PIOU is unlikely [25].

### Health system costs

Costs from the health system perspective were calculated according to economic guidelines [27, 28] and published methods [29, 30]. Health system costs considered were those incurred due to a hospitalization (including per diem costs), day surgery, outpatient visit, and emergency department visit. Costs for physician services and the proportion of medication costs covered by the public drug plan (RAMQ) were also considered. Data on hospitalizations and day surgeries were extracted from MED-ÉCHO. Ambulatory visits and drug utilization data were identified using RAMQ medical and pharmaceutical data. Unit costs were calculated for the 2013–2014 fiscal year using budget and activity reports from the Ministry of Health and Social Services (MSSS) that are submitted annually by health care institutions in Québec (AS-471) [31, 32]. Costs were converted to 2022 Canadian dollars using the Consumer Price Index (CPI) for health and personal care for the province of Québec [33]. To consider the opportunity costs associated with land and facilities being used during health care utilization, 6% was added to the unit costs for hospitalizations and surgeries and 4% for outpatient and emergency room visits [34]. Additional costs associated with security, maintenance (9.7%) [30] and building depreciation (10%) [35] were also added to all visits. The costs considered and the unit costs used (in Canadian $) are described in Additional Table 1 [32]. Unit costs were then multiplied by the number of visits (and the number of days for hospitalizations) to obtain the total costs over 3 years and the total costs per phase. The costs used in the main analyses were presented over 3 years to align with the duration of follow-up for the sample. Moreover, the phase-based costing approach was performed by first defining inflection points to represent clinically important phases of medication use among older adults [25]. Next, observation time and costs were allocated to each phase to determine the costs per phase [25]. The total costs per phase were added and divided by the duration of each phase to obtain average daily costs. The mean phase-based costs were then standardized to costs per 365 days (per annum). This novel phase-based approach has been shown to be particularly useful in dealing with censoring given that cost data are not required for all participants for the entire period of interest [25].

### Analyses

The current study analyses were guided by the conceptual framework on opioid use in older adults developed by the Agency for Healthcare Research and Quality (AHRQ), which highlights the relation between problematic opioid use and increased health service use such as emergency department visits and hospitalizations related to adverse events [36]. Descriptive analyses were conducted to describe the characteristics of the study sample. Variables from the conceptual framework that were significantly associated with both the exposure (opioid use group) and the outcome of interest (total health system costs over the 3-year follow-up period) were identified as potential confounding variables. Based on these analyses (which are not presented in full), the following potential confounding variables were used to create a probability of receiving an opioid or a potentially inappropriate prescription involving an opioid: age, physical and psychiatric multimorbidity, presence of a substance use disorder, number of daily hassles, and number of prescribers in the 3 years before opioid use or PIOU. The number of daily hassles was measured using the Daily Hassles Scale (score of 0 to 22), which includes items pertaining to family, health, finances, transportation, household chores, and social activities [37]. Substance use disorder presence was identified based on a diagnosis in the 3 years before the interview [38] or the presence of a benzodiazepine dependence based on the DSM-IV criteria [15]. Analyses were weighted using IPTW to reduce the effects of confounding and selection bias associated with receiving an opioid or an inappropriate prescription involving an opioid, or not receiving one [39]. Analyses were further adjusted for variables from the conceptual framework that had a standardized difference ≥ 10% after weights were applied [40]. These included age, sex, education level, country of origin, psychological distress, presence of a traumatic event (including an accident, natural disaster, or life-threatening disease), physical and psychiatric multimorbidity, pain severity, type of pain (inflammatory, nociceptive, neuropathic, nociplastic, other), presence of a substance use disorder, number of prescribers, continuity of care, and type of primary care practice during recruitment. Standardized differences are presented in Additional Table 2, in addition to the sample characteristics after weighting.

The Kolmogorov–Smirnov test showed that costs over the 3-year period and per phase were not normally distributed. Levene’s test showed that variances were not equal across each group (no use, opioid use, PIOU). As such, the condition of homoscedasticity was not respected. Generalized linear models (GLMs) with a gamma distribution and a log link function were chosen to study the costs associated with opioid use group (no use, opioid use, or PIOU), while accounting for the non-normal distribution of such costs [41]. In addition, several other studies have successfully modeled costs using this distribution [4, 12, 30, 42]. GLMs were conducted on a subset of 1124 individuals who had complete data. In GLMs, interactions were tested between three-level exposure (no use, opioid use, PIOU) and sex. All analyses were conducted using SPSS 27.0.

## Results

Table 1 presents the characteristics of the study sample. Further information on how characteristics were measured can be found elsewhere [15]. Exposure to opioid use or PIOU is shown to differ significantly in terms of sex, country of origin, number of daily hassles, psychological distress, multimorbidity, health service use before opioid use or PIOU, pain severity, certain types of pain (inflammatory, nociceptive, and neuropathic), presence of a substance or alcohol use disorder, number of prescribers, and type of practice. Furthermore, phase-based analyses revealed an average duration of 933 days, 210 days, and 571 days for each phase, respectively: no use, opioid use, and PIOU (Additional Table 3). The average duration of each phase is also presented by opioid use group (Additional Table 3).

**Table 1:** Characteristics of the study sample of older adults (*N* = 1201)

**Characteristics**	**By opioid use group**			*p*-value
	No use (*n* = 910)	Opioid use (*n* = 100)	PIOU (*n* = 191)	
**Patient**				
Age, n (%)				
65–74 years	583 (64.1)	66 (66.0)	111 (58.1)	0.252
75 + years	327 (35.9)	34 (34.0)	80 (41.9)	
Sex, n (%)				
Male	392 (43.1)	60 (60.0)	54 (28.3)	< 0.001^*^
Female	518 (56.9)	40 (40.0)	137 (71.7)	
Marital status, n (%)				
Married/ in common law	582 (64.2)	68 (68.0)	117 (61.6)	0.551
Single/divorced/widowed	324 (35.8)	32 (32.0)	73 (38.4)	
Education level, n (%)				
Primary	214 (23.5)	30 (30.0)	50 (26.2)	0.198
Secondary	394 (43.3)	46 (46.0)	89 (46.6)	
Post-secondary/university	302 (33.2)	24 (24.0)	52 (27.2)	
Country of origin, n (%)				
Canada	880 (96.8)	95 (95.0)	176 (92.1)	0.011^*^
Other (immigrant)	29 (3.2)	5 (5.0)	15 (7.9)	
Annual household income, n (%)				
< $25,000	282 (31.0)	28 (28.0)	71 (37.2)	0.175
≥ $25,000	628 (69.0)	72 (72.0)	120 (62.8)	
Social support index, score 0–3, mean (SD)	2.78 (SD: 0.63)	2.76 (SD: 0.67)	2.73 (SD: 0.72)	0.705
Number of daily hassles, score 0–22, mean (SD)	4.61 (SD: 3.88)	3.88 (SD: 3.66)	5.24 (SD: 3.62)	0.013*
K10 Psychological distress, score 10–50, mean (SD)	17.98 (SD: 7.01)	17.07 (SD: 6.47)	20.87 (SD: 7.83)	< 0.001^*^
Presence of traumatic events, n (%)				
Childhood	115 (12.6)	10 (10.2)	29 (15.8)	0.387
Sexual abuse, assault, violence, stalked, kidnapping, war/combat	239 (26.8)	24 (24.5)	61 (33.2)	0.165
Other (accident, natural disaster, life-threatening disease)	454 (50.9)	49 (50.0)	107 (58.2)	0.184
Multimorbidity, n (%)				
0–2 chronic physical conditions + no common mental disorder	114 (13.0)	13 (13.8)	7 (3.9)	0.001^*^
0–2 chronic physical conditions + ≥ 1 common mental disorder	35 (4.0)	2 (2.1)	5 (2.8)	
≥ 3 chronic physical conditions + no common mental disorder	467 (53.2)	61 (64.9)	96 (53.3)	
≥ 3 chronic physical conditions + ≥ 1 common mental disorder	262 (29.8)	18 (19.1)	72 (40.0)	
Health service use, mean (SD)				
Outpatient visits in 3 months before opioid use or PIOU	1.72 (SD: 1.71)	1.51 (SD: 1.19)	3.78 (SD: 5.72)	< 0.001^*^
Hospitalizations in 3 years before opioid use or PIOU	1.22 (SD: 2.00)	1.18 (SD: 2.31)	2.55 (SD: 3.04)	< 0.001^*^
Emergency department visits in 3 years before opioid use or PIOU	0.91 (SD: 1.63)	0.77 (SD: 1.44)	2.20 (SD: 2.49)	< 0.001^*^
**Pain**				
Pain severity, n (%)				< 0.001^*^
No/low pain	508 (56.8)	57 (57.6)	84 (45.4)	
Moderate pain	360 (40.3)	38 (38.4)	79 (42.7)	
Extreme pain	26 (2.9)	4 (4.0)	22 (11.9)	
Pain type, n (%)				
Inflammatory	224 (24.6)	19 (19.0)	80 (41.9)	< 0.001^*^
Nociceptive	93 (10.2)	4 (4.0)	51 (26.7)	< 0.001^*^
Neuropathic	69 (7.6)	13 (13.0)	28 (14.7)	0.003^*^
Nociplastic	50 (5.5)	1 (1.0)	12 (6.3)	0.125
Other	45 (4.9)	3 (3.0)	16 (8.4)	0.088^†^
**Substance use**				
Substance or alcohol use disorder				
Absence	837 (92.0)	98 (98.0)	168 (88.0)	0.012^*^
Presence	73 (8.0)	2 (2.0)	23 (12.0)	
Alcohol use				0.907
Non-drinkers	225 (25.3)	25 (25.3)	46 (24.9)	
Light to moderate drinkers	622 (69.9)	68 (68.7)	127 (68.6)	
Heavy drinkers	43 (4.8)	6 (6.1)	12 (6.5)	
**Provider**				
Number of distinct prescribers in 3 years before opioid use or PIOU, mean (SD)	3.45 (SD: 2.73)	3.90 (SD: 2.65)	5.88 (SD: 3.64)	< 0.001^*^
Continuity of care index, mean (SD)	0.24 (SD: 0.24)	0.19 (SD: 0.21)	0.21 (SD: 0.23)	0.083^†^
**Health care system**				
Type of practice, n (%)				
FMG or CLSC	361 (40.0)	53 (54.1)	83 (43.7)	0.024^*^
Private clinic with < 3 physicians	273 (30.3)	23 (23.5)	43 (22.6)	
Private clinic with ≥ 3 physicians	268 (29.7)	22 (22.4)	64 (33.7)	

*Abbreviations*: *CLSC* Local community service centres, *FMG* Family medicine groups, *K10* 10-item Kessler Psychological Distress Scale, *PIOU* Potentially inappropriate medication use involving opioids, *SD* Standard deviation

^†^*p* < 0.10, ^*^p < 0.05

**Fig. 1:**
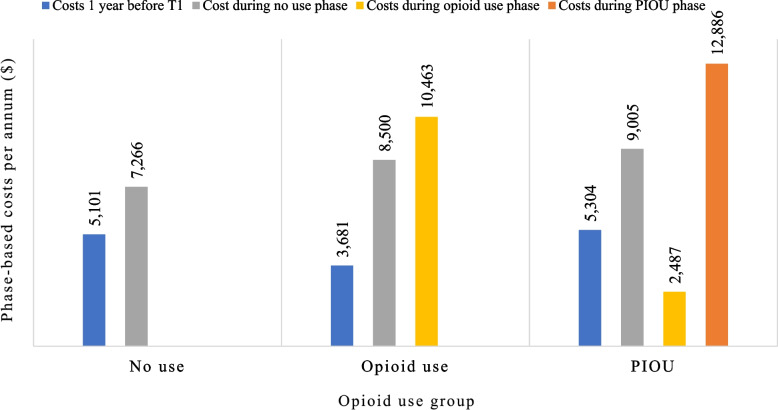
Weighted phase-based costs per annum ($) by opioid use group PIOU: Potentially inappropriate medication use involving opioids. Weighted using IPTW

Figure 1 presents the average weighted costs in the year prior to interview (T1) and the average weighted phase-based costs per 365 days by opioid use group. The descriptive findings presented in Fig. 1 show that among individuals who went on to become PIOU, health system costs incurred during the phase preceding opioid use were on average higher ($9,005) as compared to opioid users ($8,500) and non-users ($7,266) for the same phase, even after IPTW. When looking at the difference in health system costs incurred in the years following interview as compared to the preceding year (during which there was no opioid use due to a wash-out period), the results showed an average increase in costs reaching $2,165 ($7,266—$5,101), $4,819 ($8,500—$3,681), and $3,701 ($9,005—$5,304) in non-opioid users, opioid users and PIOU (Fig. 1).

Table 2 presents adjusted and weighted 3-year costs associated with opioid use and PIOU using generalized linear models. Mean adjusted 3-year costs are significantly higher in the opioid use category compared to no use (∆$2,222), in the PIOU category compared to no use (∆$8,987), and in the PIOU category compared to opioid use (∆$6,766). In general, 3-year costs were highest for medications, followed by outpatient visits. Table 2 also presents the impact of other study variables on health system costs. Of note, over the 3-year period, the presence of ≥ 3 chronic physical conditions with or without a common mental disorder were both associated with higher costs compared to 0 to 2 chronic physical conditions and no common mental disorder. Moreover, the main interaction term between opioid use categories and sex was significant in the analyses (*p* = 0.002). Effect modification by sex was observed for opioid use versus no use, as shown by the non-overlapping confidence intervals between males and females in Table 2.

**Table 2:** Total adjusted and weighted excess 3-year health system costs for opioid use and PIOU from generalized linear models (*N* = 1124)

Group comparisons	Δ Costs (95% CI)
**PIOU vs. no use**	$8,987 (CI: $7,370, $10,605)^*^
**PIOU vs. opioid use**	$6,766 (CI: $5,253, $8,278)^*^
**Opioid use vs. no use**	$2,222 (CI: $1,179, $3,264)^*^
**Males**	
***PIOU vs. no use***	$9,128 (CI: $6,743, $11,514)^*^
***PIOU vs. opioid use***	$5,009 (CI: $2,653, $7,365)^*^
***Opioid use vs. no use***	$4,119 (CI: $2,441, $5,798)^*^
**Females**	
***PIOU vs. no use***	$8,847 (CI: $7,031, $10,663)*
***PIOU vs. opioid use***	$8,258 (CI: $6,413, $10,103)^*^
***Opioid use vs. no use***	$589 (CI: –$666, $1,844)
Age 75 + vs. 65–74	$5,677 (CI: $4,438, $6,915)^*^
Male sex vs. female	$2,251 (CI: $1,210, $3,291)^*^
Primary education vs. post-secondary/university	$2,834 (CI: $1,462, $4,207)^*^
Secondary education vs. post-secondary/university	$1,813 (CI: $710, $2,916)^*^
Country of origin Canada vs. other country	$1,276 (CI: –$758, $3,310)
Presence of a traumatic life event (including an accident, natural disaster, life threatening disease)	–$561 (CI: –$1,512, $391)
≥ 3 CPC + ≥ 1 CMD vs. 0–2 CPC + no CMD	$7,996 (CI: $6,337, $9,655)^*^
≥ 3 CPC + no CMD vs. 0–2 CPC + no CMD	$8,433 (CI: $6,826, $10,039)^*^
0–2 CPC + ≥ 1 CMD vs. 0–2 CPC + no CMD	$1,423 (CI: –$924, $3,769)
Extreme pain severity vs. no/low pain	$1,534 (CI: –$710, $3,777)
Moderate pain severity vs. no/low pain	$1,180 (CI: $187, $2,173)*
Presence of inflammatory pain vs. absence	$1,103 (CI: –$37, $2,243)
Presence of nociceptive pain vs. absence	–$523 (CI: –$1,959, $914)
Presence of neuropathic pain vs. absence	–$939 (CI: –$2,455, $578)
Presence of nociplastic pain vs. absence	–$2,475 (CI: –$4,699, –$251)^*^
Presence of other pain vs. absence	$2,606 (CI: $139, $5,074)^*^
Presence substance or alcohol use disorder vs. absence	$5,405 (CI: $3,424, $7,386)^*^
Recruited in private clinic with < 3 physicians vs. FMG or CLSC	$2,798 (CI: $1,540, $4,056)^*^
Recruited in private clinic with ≥ 3 physicians vs. FMG or CLSC	$2,113 (CI: $935, $3,291)^*^

Costs presented for 2022. Weighted using IPTW and adjusted for age, sex, education level, country of origin, psychological distress, presence of a traumatic event (including an accident, natural disaster, or life-threatening disease), physical and psychiatric multimorbidity, pain severity, type of pain (inflammatory, nociceptive, neuropathic, nociplastic, other), presence of a substance use disorder, number of prescribers, continuity of care, and type of practice during recruitment

*Abbreviations*: *CLSC* Local community service centres, *CMD* Common mental disorder, *CPC* Chronic physical condition, *FMG* Family medicine groups, *PIOU* Potentially inappropriate medication use involving opioids

^*^p < 0.05

Unweighted and weighted phase-based costs (per annum) are presented in Additional Table 4. These descriptive findings show that the highest health system costs were incurred by participants in the PIOU phase, followed by the no-use and opioid use phase. Additional Table 4 also presents the proportion of phase-based costs by type of health service used. In the opioid use and PIOU phases, outpatient visits accounted for the greatest proportion of costs, whereas in the non-use phase, hospitalizations accounted for the largest proportion of costs.

## Discussion

In the current study, opioid use and PIOU compared to no use were associated with additional 3-year mean costs of $2,222 and $8,987, respectively. Studies on the costs associated with PIOU in older adults have found excess mean costs ranging between $1,526 and $4,976 US annually [10–12]. In a study on patients with osteoarthritis taking opioids, the 6-month excess mean costs associated with a potential drug interaction involving an opioid compared to no potential interaction reached $1,207 US (*p* = 0.001) [10]. The higher costs were attributable to hospitalizations and costs for medications, including both opioid and non-opioid medications [10]. In a similar study, the same authors focused on older adults with chronic low back pain using opioids and reported higher mean costs over a 6-month period reaching $763 US (*p* = 0.013) in those exposed to a potential drug-drug interaction involving an opioid compared to those without a potential interaction but exposed to an opioid [11]. The costs were predominantly driven by primary care visits and costs for medication use [11]. In both studies, exposure to a drug interaction was defined as at least 1 day of overlapping opioid use with another drug metabolized by the cytochrome P450 (CYP) enzyme system [10, 11]. The authors evaluated costs from the payer perspective, which included outpatient procedures, hospitalizations, primary care visits, emergency room visits, and costs for medications [10, 11]. Another study in the United States on patients aged 65 years and older reported higher mean total health care costs reaching $4,976 US (*p* = 0.03) over a 360-day period among those exposed to PIOU compared to those who were not exposed [12]. Although costs were mostly driven by medical costs, the authors do not provide further breakdown of costs by type of health service used [12]. PIOU was defined as the use of pentazocine or meperidine, considered high-severity narcotics according to the Beers criteria [12, 43].

The present study reports on the health system costs over a 3-year period associated with opioid use and PIOU in a non-cancer older adult population. We found that costs were significantly higher in phases involving opioid use and PIOU. In the PIOU phase, costs were mostly driven by outpatient visits and hospitalizations rather than medication-related costs, which is concordant with the literature [10–12]. In general, higher health service use may reflect unmet needs for emotional or mental health conditions or substance use problems, which has been linked with a higher prevalence of problematic opioid use in Canadian adults [44], and a deterioration in self-reported physical health [45]. This is further supported by our findings that physical and psychiatric conditions, and the presence of a substance use disorder, are factors associated with PIOU [15], which are significantly driving health system costs, among other factors, in this sample of older adults. Another potential explanation is that inadequate pain management drives the association between PIOU and increased costs due to increased health service utilization among older adults [46]. This relationship, however, may also be circumstantial since, to receive an opioid prescription, one needs to visit a physician more regularly, which would increase health service use and associated costs.

When studying the costs in the year prior to study recruitment when individuals had no opioid use, those who eventually went on to develop PIOU incurred higher costs related to health service utilization as compared to those who were opioid users and those who never went on to use an opioid during follow-up. Older adults with PIOU may be high users of the health care system and incur higher costs due to underlying factors such as socioeconomic and mental health conditions or substance use disorders [47], which have been associated with PIOU [15]. Among individuals with PIOU, costs incurred during the opioid use phase were the lowest compared to other phases, with an average of 21 days spent in this phase, indicating that these individuals were very quick to be classified as PIOU. This suggests that the window of opportunity for preventing individuals from receiving medications leading to PIOU may be small, and that prescribers should intervene early to prevent PIOU from developing in older adults. In fact, safer prescribing of opioids should be encouraged altogether.

The current findings showed that the excess 3-year costs associated with PIOU compared to no use reached $8,987 CAD, after controlling for patient and health system variables from the conceptual framework, including important mental health variables [36]. The excess 3-year costs associated with opioid use as compared to no use reached $2,222 CAD, whereas those associated with PIOU compared to opioid use reached $6,766 CAD. If one were to consider purchasing power parity [48], the average estimates observed for PIOU compared to opioid use are similar to those reported in the literature, ranging between $4,578 and $7,242 US per 3 years [10, 11]. The estimates observed in the current study for PIOU compared to no use are lower than previous reports estimating costs at $14,928 US per 3 years [12]. Differing costs may be due to the failure of previous studies to account for significant mental health comorbidities such as substance use disorders. Another possible reason for the discrepancies in cost estimates could be the inclusion of older adults with cancer, which may have resulted in higher costs associated with cancer care [12].

This study has many strengths that should be highlighted. First, we used a phase-based costing approach that allowed us to estimate costs over the opioid use trajectory. Costs were extrapolated to 2022 to reflect the current economic impact on the health care system. Another methodological strength was the use of IPTW to reduce confounding and selection biases. In fact, we were able to control for several important variables by linking self-reported health survey data with administrative medical data. Provincial administrative data are known to provide accurate information on health service use and dispensed prescriptions, given that older adults are covered by the public drug insurance plan as of age 65 and that the Québec population has universal health coverage. In addition, we implemented a 1-year wash-out period to reduce the potential for prevalent user bias associated with opioid use, namely, that participants who have received an opioid in the past may be more resilient to its adverse effects [49, 50]. These findings will help inform decision makers on the economic impact of PIOU from a health system perspective to improve the allocation of resources for pain management and safer opioid use in older adults.

Certain limitations must also be considered when interpreting the findings. For instance, the generalizability is limited given that findings pertained to community-dwelling older adults consulting in primary care and universally covered by the Québec public health care system. As such, cost analyses cannot be extended to institutionalized older adults or those without access to a primary care physician, nor can they be generalized to other health care systems that do not offer universal access to health care. In addition, although unit costs were converted with the CPI to 2022 dollars, they were calculated based on budget reports from 2013–2014, which may limit the external validity of the findings to the present day. Future research could benefit from studying opioid use and associated health service utilization patterns using data from the current context, especially given the increased attention to opioid-related concerns in recent years and changes in prescribing practices. Medication use was measured using prescription claims data which inform on medications obtained in the community but not on medications provided during hospitalizations. Moreover, medications obtained without a prescription, not covered by the public drug plan, and exact medication consumption reflecting patient adherence are not registered in the administrative databases. Administrative databases are also subject to information biases from coding errors as they were conceived for administrative and billing purposes. However, Québec administrative pharmaceutical data are deemed a valid source for identifying drugs dispensed to the population [51]. Finally, the costs related to homecare and long-term care could not be considered. Although this might lead to an underestimation of health system costs, we do not expect it to be significant given that older adults in our sample were recruited in primary care practices and were residing at home when the interviews were conducted.

## Conclusions

The present findings suggest that opioid use and potentially inappropriate medication use involving opioids in older adults are associated with considerable health system costs. Underlying health conditions may be contributing to opioid use and potentially inappropriate medication use involving opioids, ultimately resulting in increased costs related to health service utilization. Better use of health resources should be considered to treat pain in older adults, such as considering non-pharmacological alternatives and the coordinated treatment of pain and mental/physical health comorbidities including substance use disorders. Future research would benefit from evaluating costs from the patient perspective to gain a societal understanding of the impact of potentially inappropriate medication use involving opioids in older adults.

### Supplementary Information

**Additional file 1: Table 1. **Costs considered according to the health system perspective [32].

**Additional file 2: Table 2.** Characteristics of the study sample after inverse probability of treatment weighting.

**Additional file 3: Table 3.** Average duration of each exposure phase overall and by opioid use group.

**Additional file 4: Table 4. **Mean phase-based costs per annum ($) by type of health service according to exposure phase.

## Data Availability

The datasets generated and/or analysed during the current study are not publicly available due to privacy and ethical restrictions related to the use of provincial health administrative data. In addition, participants were not asked to give their informed consent for data sharing. The authors are therefore not legally authorized to share or publicly publish linked provincial health and administrative medical record data. All data requests can be made to *CIUSSS-Estrie CHUS* ethics committee.

## Abbreviations

CHUS: Centre Hospitalier de Sherbrooke
CI: Confidence interval
CLSC: Local community service centre
CNS: Central nervous system
CPI: Consumer Price Index
CYP: Cytochrome
DSM-IV: Diagnostic and Statistical Manual of Mental Disorders, fourth edition
FMG: Family medicine groups
GLM: Generalized linear model
ICD: International Classification of Diseases
IPTW: Inverse probability of treatment weight
K10: 10-Item Kessler Psychological Distress Scale
MED-ÉCHO: Maintenance et exploitation des données pour l’étude de la clientèle hospitalière
MSSS: Ministry of Health and Social Services
PIOU: Potentially inappropriate medication use involving opioids
RAMQ: Régie de l’assurance maladie du Québec
SD: Standard deviation
