# Supplementary material for: Health care system costs related to potentially inappropriate medication use involving opioids in older adults in Canada
Source: BMC Health Serv Res. 2023 Nov 24;23:1295. doi: 10.1186/s12913-023-10303-2 (PMC10668473; doi:10.1186/s12913-023-10303-2)
Supplement: Supplementary file 1 — Additional file 1: Table 1. Costs considered according to the health system perspective [32]. [file 12913_2023_10303_MOESM1_ESM.docx]

**Additional Table 1: Costs considered according to the health system perspective** (32)

| **Type of health service** | **Unit of measure and description of costs** | **Additional costs considered** | **Data sources** | **Unit costs adjusted to 2022** |
| --- | --- | --- | --- | --- |
| **Emergency department visits** | - Cost per emergency department visit | - 4 % opportunity costs (34) - 9.7 % for overhead costs including security and maintenance (30) - 10 % for building depreciation costs (35) | RAMQ medical services file,  AS-471, AS-478 reports | $463.19 per emergency department visit |
| **Outpatient visits** | - Cost per outpatient visit | - 4 % opportunity costs (34) - 9.7 % for overhead costs including security and maintenance (30) - 10 % for building depreciation costs (35) | RAMQ medical services file,  AS-471, AS-478 reports | $150.92 per outpatient visit |
| **Inpatient visits including hospitalizations and day surgeries** | - Hospitalizations (cost per day + cost per hospitalization) - Day surgeries (cost per surgery) | - 6 % opportunity costs (34) - 9.7 % for overhead costs including security and maintenance (30) - 10 % for building depreciation costs (35) | MED-ÉCHO database,  AS-471, AS-478 reports | $688.33  per day, $747.92 per hospitalization  $2,206.13 per day surgery |
| **Physician billing fees** | - Fees billed to the RAMQ | - | RAMQ medical services database | - |
| **Medications** | - Portion of fees paid by the public drug plan (RAMQ) | - | RAMQ pharmaceutical services database | - |
