# Supplementary material for: Health care system costs related to potentially inappropriate medication use involving opioids in older adults in Canada
Source: BMC Health Serv Res. 2023 Nov 24;23:1295. doi: 10.1186/s12913-023-10303-2 (PMC10668473; doi:10.1186/s12913-023-10303-2)
Supplement: Supplementary file 2 — Additional file 2: Table 2. Characteristics of the study sample after inverse probability of treatment weighting. [file 12913_2023_10303_MOESM2_ESM.docx]

**Additional Table 2: Characteristics of the study sample after inverse probability of treatment weighting**

| **Characteristics** | | | **By opioid use group** | | | | p-value | Standardized difference (opioid vs no use) | Standardized difference (PIOU vs no use) |
| --- | --- | --- | --- | --- | --- | --- | --- | --- | --- |
|  | | | No use  (n = 1214) | Opioid use  (n = 995) | | PIOU  (n = 891) |  |  |  |
| **Patient** | Age, n (%) | |  |  | |  |  |  |  |
|  |  | 65-74 years | 775 (63.8%) | 630 (63.3%) | | 503 (56.5%) | 0.001* | 0.0208 | 0.1436 |
|  |  | 75+ years | 439 (36.2%) | 366 (36.7%) | | 387 (43.5%) |  |  |  |
|  | Sex, n (%) | |  |  | |  |  |  |  |
|  |  | Male | 509 (41.9%) | 526 (52.9%) | | 295 (33.1%) | <0.001* | 0.2217 | -0.1858 |
|  |  | Female | 705 (58.1%) | 469 (47.1%) | | 595 (66.9%) |  |  |  |
|  | Marital status, n (%) | |  |  | |  |  |  |  |
|  |  | Married/ in common law | 769 (63.5%) | 644 (64.7%) | | 576 (65.8%) | 0.543 | 0.0208 | 0.0417 |
|  |  | Single/divorced/widowed | 442 (36.5%) | 351 (35.3%) | | 299 (34.2%) |  |  |  |
|  | Education level, n (%) | |  |  | |  | 0.004* | -0.1481 | -0.1082 |
|  |  | Primary | 285 (23.5%) | 281 (28.2%) | | 235 (26.4%) |  |  |  |
|  |  | Secondary | 530 (43.7%) | 451 (45.3%) | | 414 (46.5%) |  |  |  |
|  |  | Post-secondary/university | 399 (32.9%) | 263 (26.4%) | | 241 (27.1%) |  |  |  |
|  | Country of origin, n (%) | |  |  | |  |  |  |  |
|  |  | Canada | 1176 (96.9%) | 965 (96.9%) | | 795 (89.2%) | <0.001* | 0 | -0.3318 |
|  |  | Other (immigrant) | 38 (3.1%) | 31 (3.1%) | | 96 (10.8%) |  |  |  |
|  | Annual household income, n (%) | |  |  | |  |  |  |  |
|  |  | < $25,000 | 384 (31.6%) | 276 (27.7%) | | 313 (35.2%) | 0.002* | 0.0874 | -0.0638 |
|  |  | ≥ $25,000 | 830 (68.4%) | 719 (72.3%) | | 577 (64.8%) |  |  |  |
|  | Social support index, score 0-3, mean (SD) | | 2.84 (0.48) | 2.84 (0.48) | | 2.86 (0.46) | 0.488 | 0 | 0.0423 |
|  | Number of daily hassles, score 0-22, mean (SD) | | 4.90 (3.97) | 4.77 (4.45) | | 4.74 (3.25) | 0.579 | -0.0310 | -0.0435 |
|  | K10 Psychological distress, score 10-50, mean (SD) | | 18.21 (7.10) | 17.47 (6.21) | | 19.40 (6.89) | <0.001* | -0.1102 | 0.1698 |
|  | Presence of traumatic events, n (%) | |  |  | |  |  |  |  |
|  |  | Childhood | 158 (13.0%) | 99 (9.9%) | | 116 (13.1%) | 0.046* | -0.0936 | 0 |
|  |  | Sexual abuse, assault, violence, stalked, kidnapping, war/combat | 328 (27.0%) | 245 (24.6%) | | 241 (27.2%) | 0.340 | -0.0456 | 0 |
|  |  | Other (accident, natural disaster, life-threatening disease) | 626 (51.6%) | 537 (53.9%) | | 523 (59.0%) | 0.003* | 0.0400 | 0.1409 |
|  | Multimorbidity, n (%) | |  |  | |  |  |  |  |
|  |  | 0-2 chronic physical conditions + no common mental disorder | 134 (11.0%) | 133 (13.4%) | | 95 (10.7%) | 0.026* | 0.1098 | 0 |
|  |  | 0-2 chronic physical conditions + ≥ 1 common mental disorder | 40 (3.3%) | 51 (5.1%) | | 25 (2.8%) |  |  |  |
|  |  | ≥ 3 chronic physical conditions + no common mental disorder | 648 (53.4%) | 525 (52.7%) | | 495 (55.6%) |  |  |  |
|  |  | ≥ 3 chronic physical conditions + ≥ 1 common mental disorder | 392 (32.3%) | 287 (28.8%) | | 276 (31.0%) |  |  |  |
|  | Health service use, mean (SD) | |  |  | |  |  |  |  |
|  |  | Outpatient visits in 3 months before opioid use or PIOU | 1.78 (1.76) | 1.66 (1.27) | | 3.44 (6.07) | <0.001* | -0.0768 | 0.3980 |
|  |  | Hospitalizations in 3 years before opioid use or PIOU | 1.39 (2.18) | 1.38 (2.81) | | 2.06 (2.68) | <0.001* | 0.2783 | -0.004 |
|  |  | Emergency department visits in 3 years before opioid use or PIOU | 1.08 (2.01) | 0.71 (1.29) | | 1.63 (1.93) | <0.001* | -0.2145 | 0.2780 |
| **Pain** | Pain severity, n (%) | |  |  | |  | <0.001* | -0.0175 | -0.1858 |
|  |  | No/low pain | 675 (55.8%) | 564 (56.7%) | | 448 (50.6%) |  |  |  |
|  |  | Moderate pain | 503 (41.6%) | 380 (38.2%) | | 361 (40.8%) |  |  |  |
|  |  | Extreme pain | 32 (2.6%) | 51 (5.1%) | | 76 (8.6%) |  |  |  |
|  | Pain type, n (%) | |  |  | |  |  |  |  |
|  |  | Inflammatory | 312 (25.7%) | 201 (20.2%) | | 321 (36.0%) | <0.001* | -0.1425 | 0.2194 |
|  |  | Nociceptive | 135 (11.1%) | 44 (4.4%) | | 200 (22.4%) | <0.001* | -0.2585 | 0.3045 |
|  |  | Neuropathic | 94 (7.7%) | 102 (10.3%) | | 118 (13.2%) | <0.001* | 0.0704 | 0.1669 |
|  |  | Nociplastic | 74 (6.1%) | 14 (1.4%) | | 38 (4.3%) | <0.001* | -0.2580 | -0.0893 |
|  |  | Other | 64 (5.3%) | 17 (1.7%) | | 50 (5.6%) | <0.001* | -0.1597 | -0.0465 |
| **Substance use** | Substance or alcohol use disorder | |  |  | |  |  |  |  |
|  |  | Absence | 1095 (90.2%) | 921 (92.6%) | | 817 (91.8%) | 0.127 | 0.1065 | 0.0695 |
|  |  | Presence | 119 (9.8%) | 74 (7.4%) | | 73 (8.2%) |  |  |  |
|  | Alcohol use | |  |  | |  | 0.007* | 0.0892 | -0.0691 |
|  |  | Non-drinkers | 321 (26.5%) | 295 (29.6%) | | 209 (23.5%) |  |  |  |
|  |  | Light to moderate drinkers | 838 (69.1%) | 640 (64.3%) | | 624 (70.0%) |  |  |  |
|  |  | Heavy drinkers | 54 (4.5%) | | 60 (6.0%) | 58 (6.5%) |  |  |  |
| **Provider** | Number of distinct prescribers in 3 years before opioid use or PIOU, mean (SD) | | 3.97 (3.16) | | 4.14 (2.98) | 4.41 (2.60) | 0.004* | 0.0551 | 0.1497 |
|  | Continuity of care index, mean (SD) | | 0.23 (0.24) | | 0.18 (0.19) | 0.24 (0.26) | <0.001* | -0.2283 | 0.0471 |
| **Health care system** | Type of practice, n (%) | |  | |  |  |  |  |  |
|  |  | FMG or CLSC | 484 (40.0%) | | 528 (53.1%) | 366 (41.1%) | <0.001* | 0.2425 | 0.0734 |
|  |  | Private clinic with < 3 physicians | 358 (29.6%) | | 231 (23.2%) | 228 (25.6%) |  |  |  |
|  |  | Private clinic with ≥ 3 physicians | 369 (30.5%) | | 236 (23.7%) | 297 (33.3%) |  |  |  |

Abbreviations: CLSC, Local community service centres; FMG, Family medicine groups; K10, 10-item Kessler Psychological Distress Scale; PIOU, Potentially inappropriate opioid use; SD, Standard deviation

*p < 0.05
