# Supplementary material for: Health care system costs related to potentially inappropriate medication use involving opioids in older adults in Canada
Source: BMC Health Serv Res. 2023 Nov 24;23:1295. doi: 10.1186/s12913-023-10303-2 (PMC10668473; doi:10.1186/s12913-023-10303-2)
Supplement: Supplementary file 3 — Additional file 3: Table 3. Average duration of each exposure phase overall and by opioid use group. [file 12913_2023_10303_MOESM3_ESM.docx]

**Additional Table 3: Average duration of each exposure phase overall and by opioid use group**

|  | **Average duration of each exposure phase (in days)** | | |
| --- | --- | --- | --- |
|  | **No use phase** | **Opioid use phase** | **PIOU phase** |
| **Overall** | 933 (range: 1-1095, median: 1095) | 210 (range: 1-1089, median: 1) | 571 (range: 2-1094, median: 611) |
| **By opioid use group** | | | |
| **No use (n = 910)** | 1075 (range: 7-1095, median: 1095) | - | - |
| **Opioid use (n = 100)** | 519 (range: 8-1095, median: 492.5) | 570 (range: 1-1089, median: 604.5) | - |
| **PIOU (n = 191)** | 472 (range: 1-1079, median: 444) | 21 (range: 1-806, median: 1) | 571 (range: 2-1094, median: 611) |
