# Supplementary material for: Health care system costs related to potentially inappropriate medication use involving opioids in older adults in Canada
Source: BMC Health Serv Res. 2023 Nov 24;23:1295. doi: 10.1186/s12913-023-10303-2 (PMC10668473; doi:10.1186/s12913-023-10303-2)
Supplement: Supplementary file 4 — Additional file 4: Table 4. Mean phase-based costs per annum ($) by type of health service according to exposure phase. [file 12913_2023_10303_MOESM4_ESM.docx]

**Additional Table 4: Mean phase-based costs per annum ($) by type of health service according to exposure phase**

| **Type of health service** | | **Mean unweighted costs by phase**  **(Per annum)** | | | **Mean weighted costs by phase**  **(Per annum)** | | |
| --- | --- | --- | --- | --- | --- | --- | --- |
|  |  | **Exposure phase** | | | | | |
|  |  | **No use** | **Opioid use** | **PIOU** | **No use** | **Opioid use** | **PIOU** |
| **Emergency department costs** | | 279 (3.8%) | 299 (4.9%) | 846 (5.7%) | 320 (3.9%) | 319 (4.8%) | 556 (4.7%) |
| **Outpatient costs** | | 1,772 (23.8%) | 2,653 (43.8%) | 5,506 (37.2%) | 1,732 (21.2%) | 2,997 (44.7%) | 5,357 (45.6%) |
| **Inpatient costs** | Hospitalizations | 2,186 (29.4%) | 1,493 (24.7%) | 4,912 (33.2%) | 2,905 (35.6%) | 1,521 (22.7%) | 2,738 (23.3%) |
|  | Day surgeries | 288 (3.9%) | 456 (7.5%) | 522 (3.5%) | 261 (3.2%) | 402 (6.0%) | 601 (5.1%) |
| **Physician billing fees** | | 1,688 (22.7%) | 1,078 (17.8%) | 2,741 (18.5%) | 2,183 (26.7%) | 1,360 (20.3%) | 2,207 (18.8%) |
| **Medications** | | 1,216 (16.4%) | 70 (1.2%) | 275 (1.9%) | 760 (9.3%) | 99 (1.5%) | 279 (2.4%) |
| **Total health system costs** | | 7,430 | 6,050 | 14,802 | 8,162 | 6,697 | 11,738 |

Weighted using IPTW
